# Soft Gold Foil

**Published:** 1887-12

**Authors:** Geo. H. Winkler


ARTICLE III.

### SOFT GOLD FOIL.

GEO. H. WINKLER, M. D., D. D. S.

[Read before the Southern Dental Association at Old Point Comfort, Va., September 1st, 1887.]

In offering this essay on gold foil, I shall confine myself to a discussion of that form of foil which is rendered non-cohesive by the manufacturers, through methods unnecessary to relate, whose surfaces are deprived by various means of that inherent property of chemically pure gold, freshly annealed, of welding or cohering when pressed or malleted together, and known under the name of soft gold foil. I propose to elucidate as clearly and concisely as I possibly can my appreciation of the intrinsic value of soft gold foil in the preservation of teeth, the character of the operations in which its use is specially indicated, and my method of using it. In 1857 Dr. Robert Anther, of Baltimore, Md., published a treatise on what was then called adhesive foil. His views and ideas expressed in that paper met with considerable opposition from the profession at large, and it was a number of years before cohesive foil came into general use; and up to that time soft foil was the only gold inserted in teeth.

The older members of our profession are well aware of the fact that teeth were well preserved then as they are now by its use. I, myself, have seen fillings of soft gold foil inserted in the mouths of patients in Charleston, S. C., by Drs. Brewster, Houston, Manifeldt, and my father-in-law, Dr. J. B. Patrick, that were still preserving the teeth after a duration of 30 to 40 years. Some of the fillings were so soft that an excavator could be readily pressed through them, and yet the cavities they occupied were entirely free from decay.

In a practice extending over nineteen years I have been in the habit of using soft gold foil, cohesive foil and a combination of the two, selecting that kind which, in my judgment, seemed best adapted to each case presented, and I am satisfied there is no gold so entirely satisfactory as soft gold foil, both to operator and patient, in cavities where I use it. It preserves the teeth at least as lastingly as any other gold; it is always used honestly for the preservation of teeth, and never for show; it can be inserted with the greatest ease; it can be introduced rapidly—so rapidly in fact that a suitably prepared cavity can be thoroughly plugged with soft foil in from five to ten minutes that would require nearly or quite one hour to fill with cohesive gold; it is the most expeditious method of plugging with gold known to human skill; it is readily adapted to the walls of the cavity; it makes, when properly condensed, a filling sufficiently compact and hard for any service that may be required of it; it expands laterally under pressure exerted on the surface of a filling when condensing, thus rendering more certain the hermetic sealing of the cavity; the process of its insertion in a cavity places always a comparatively thick pad of gold against the wall from which the cavity is filled and between it and the instrument thus protecting that wall from being bruised or crumbled by the tempered steel. This condition alone makes it extremely valuable at the cervical walls of approximal cavities of all teeth, and most decidedly so in those of soft texture. Soft foil can be successfully introduced into cavities difficult of access without a sacrifice of sound tooth structure; it enables an operator to economize greatly in time and labor, and protects his patients from fatigue and in many operations from utter exhaustion. On this account it is especially grateful to frail persons or little children. It does away with the necessity of using the rubber dam, which is objectionable to many; it can be filled and finished with great facility and with absolute certainty, especially at the cervical margins of approximal cavities; no overhanging edges of gold are ever left at these points in soft foil fillings. The habitual use of it induces the highest appreciation of the great value and genuine merit of cohesive foil in operations requiring the use of the latter; the proper and successful handling of it seems to affect and develop manual dexterity, for nearly all soft foil operators are skillful manipulators of cohesive gold. The reason for this excellence is apparent, for soft foil operators depend entirely for success in their work upon the mechanical principles of mortising and dovetailing and their mechanical ingenuity is thus constantly exercised and trained, and they bring this skill of mind and hand to bear upon their cohesive foil operation. For the foregoing reasons I believe soft foil to be unquestionably the very best preparation of gold for filling the following cavities in teeth: All simple cavities—by which term I mean those cavities whose walls are intact, situated upon the grinding, buccal, palatal or labial surfaces of teeth, excepting always such as are extremely small or as are in the form of narrow fissures (I fill these with cohesive foil;) cavities occurring at or under the free margin of the gums—these cavities can be filled and condensed so rapidly that the mucus secretion has not time to dampen the filling, the first pellet or two sometimes actually acting as a compress on the gum, effectually protecting the balance of the filling during insertion from its secretion; simple approximal cavities, rendered accessible through separators, wedges or cutting instruments, and not requiring to be knuckled up; approximal cavities of bicuspids or molars when the crown of the tooth is involved by having crushed through to the decay beyond or by having been cut out by the operator, the buccal and palatal walls of the tooth remaining intact. In such cavities as these, which are partly contour, the approximal portion of the cavities should be filled from the cervical wall for at least two-thirds of their extent with soft foil · the contoured portion and crown of the filling should be of cohesive gold welded to the already well-condensed soft, and made more secure still, if necessary, by suitable undercuts. Crown and buccal cavities, or crown and palatal cavities, connected by a narrow fissure, should be filled in the crown and buccal or crown and palatal with soft foil, and the fissure should be built up with cohesive foil welded to each of the two fillings, and in such cases rendered more secure by proper undercuts.

In fact, I seldom fail even in contour work to begin each filling with one or more pellets of soft foil. The certainty with which cohesive gold can be welded to soft by applying small pieces of the former, fresh from the lamp, to the latter, and the remarkable strength of the union, is an incredible fact to those who have never used or seen the combination.

I now come to the method of insertion and condensation. Soft foil is used in the form of ropes, ribbons, cylinders and pellets. I prefer the pellets, and use them almost exclusively, for the reason that they are easy to make and convenient to fold into the cavity ; they are readily guided into their position, and there is nothing about them which interferes with an unobtrusive view of the work in hand ; and then, I think a pellet can be condensed into a more compact mass than either a rope, ribbon or cylinder. In preparing my pellets I take a small piece of foil torn from the leaf larger or smaller, according to the size of the cavity to be filled, and fold it upon itself lightly, first in one direction and then crosswise, until I have a small, loosely-folded mat which I crumple together and roll between my fingers and thumb into an oblong pellet, having one end more pointed than the other. The pointed end serves to guide the pellet to its place when being inserted, and the crumpling of it forms on its every fold innumerable minute wrinkles, some salient, others reentrant, which under pressure interdigitate so thoroughly that the finished plug is held together with the tenacity almost of a solid mass. This result is accomplished by the interdigitations of the salient and reentrant angles of the crumpled pellet. The interdigitation of the crystals of the foil is, in my opinion, a myth, because I have proved to my own satisfaction that a sheet of foil folded upon itself to a strip, and that strip rolled upon itself into a cylinder, and then compressed between pluggers into a hard mat, although full of crystals, is capable of being carefully unfolded into foil again ; but if a similar sheet of foil be crumpled and rolled into a pellet and then compressed, there is no amount of skill or care that can ever unfold it again. The preparation of my cavity is not dissimilar to the usual form, except that I depend almost entirely upon undercuts, having, of course, two opposite each other, and seldom resort to retaining pits. I insert the first pellet of gold against the cervical wall in approximal cavities, and against the distal wall in nearly all others, allowing part of it to lie along the bottom of the cavity like a foot piece, and condensing it all with my plugging instruments, so that it shall conform in shape to the undercut wall against which it is placed a small portion of it left protruding from the cavity to form a surplus; then remaining pellets are then inserted and condensed as before, each preserving as nearly as possible the general conformation of the first until the last piece is crowded to its place against the side of the cavity nearest or most convenient to me. The last pellet of gold should be inserted always at the side or edge of the cavity, The gold slips or glides over the edge of the cavity more perfectly than over a surface of partly condensed gold, such as is found where an excavator is driven into a filling and pressed laterally to make room for more gold. The gold is thus laterally dovetailed into the cavity. The surplus is now pressed together in the form of a cone, and the whole mass is then still further condensed. Some of the instruments used for folding the foil into a cavity have smooth or very slightly serated points; others have deep serations, while others still are so sharp as to be denominated needle points. I prefer the smooth, and exercise the greatest care to avoid punching my instruments through the foil. Every puncture made by deeply serated or needle pointed instruments through soft gold foil in folding it into the cavity assists in diminishing the integrity of the filling, and even the habit of pressing an excavator into a partly condensed filling in order to make room for more gold to be inserted, as is done by some operators, is false in principle and frequently unsuccessful in practice. If a cavity is found to be not full enough, the gold in it should be thoroughly condensed and cohesive foil welded on to complete the filling. In folding and condensing the foil into a tooth, I use hand pressure, automatic mallets and the lead mallets; in finally condensing the filling, I use heavy instruments of various shapes (which it would be unprofitable to describe here) for hand pressure, and a few suitably formed instruments with the lead mallet, but I depend principally upon plugging forceps for condensing the gold in buccal, palatal, labial and approximal cavities, and in some cases the fillings upon the crowns of low molars. These forceps are adapted to the various positions of the plugs they are intended to condense. The last mentioned, those for condensing crown cavities in lower molars, being so formed that one beak of them bears on the back of a pad placed outside of the mouth under the jaw and beneath the tooth operated upon, while the other beak forms a condensing point and is pressed upon the gold in the tooth. I use these forceps with great satisfaction upon little children and very timid persons. I have another instrument for condensing the gold in crown cavities, which I call a biting instrument—to a heavy handle is fitted a steel disk, on one side of which is soldered a pad of block tin; to the other is screwed a steel plugging point. The point is placed upon the gold and moved on it from place to place, the patient biting upon the tin pad above and below, as the case might be, each time the instrument is moved, until the entire plug is thoroughly condensed under the powerful pressure of the muscles of the jaw.

By carefully noting the above explanation of my method of filling with soft foil, it will be observed that having a cavity properly prepared for plugging, I am governed in the prosecution of my work by three rules or laws, which I regard as absolutely essential to an exhibition of that high excellence of attainment possible, and that should be secured in soft foil operations. First, each pellet of foil is so folded into the cavity the completed mass forms a perfect dovetailed mortise, and depends entirely upon the mechanical principle of dovetailing for its retention in the cavity. Second, each pellet of foil, as well as the entire mass, is most carefully guarded from being punctured or torn by the instruments, in order that the strongest integrity of the gold remain at its maximum: and third, a final, complete and powerful condensation of the filling, by means of instruments ingeniously adapted to their work and made according to those laws of mechanism, which afford readily, absolutely and safely, the important power needed. The filing off and polishing of soft foil fillings is similar to the finishing of other plugs.

I use, in my practice, Nos. 3, 5 and 6 gold foil, because they seem most perfectly to combine that thickness which gives strength with that pliability which ensures successful manipulation; and I am extremely careful in selecting my foil to avoid those makes which are apparently weak or rotten in their texture.

I expect that some of the statements in my paper will be received with scepticism by operators who do not use soft gold foil, but I am perfectly aware that there is not a single statement contained in it which is not capable of the clearest proof by actual demonstration.

The manipulation of soft gold foil is a beautiful art in itself, entirely distinct and different from that art which is invoked in cohesive foil operations. I think every operator should perfect himself in both arts, and every one who fails to do so deprives himself of many superior advantages in operating, that he would otherwise enjoy, and withholds from his patients, in many cases, great relief from fatigue.—*Southern Dental Journal.*

